# Representational dynamics of the main dimensions of object space: Face/body selectivity aligns temporally with animal taxonomy but not with animacy

**DOI:** 10.1167/jov.25.13.2

**Published:** 2025-11-03

**Authors:** Gaëlle Leys, Chiu-Yueh Chen, Andreas von Leupoldt, J. Brendan Ritchie, Hans Op de Beeck

**Affiliations:** 1 Research Unit Brain & Cognition, KU Leuven, Leuven, Belgium; 2 Centre de Recherche en Neurosciences de Lyon Inserm U1028, Lyon, France; 3 Research Group Health Psychology, KU Leuven, Leuven, Belgium; 4 Leuven Brain Institute, Leuven, Belgium; 5 Department of Neuroscience, University of Lethbridge, Lethbridge, Canada

**Keywords:** object perception, faces & bodies, representational dynamics

## Abstract

Object representations are organized according to multiple dimensions, with an important role for the distinction between animate and inanimate objects and for selectivity for faces versus bodies. For other dimensions, questions remain how they stand relative to these two primary dimensions. One such dimension is a graded selectivity for the taxonomic level that an animal belongs to. Earlier research suggested that animacy can be understood as a graded selectivity for animal taxonomy, although a recent functional magnetic resonance imaging study suggested that taxonomic effects are instead due to face/body selectivity. Here we investigated the temporal profile at which these distinctions emerge with multivariate electroencephalography (*N* = 25), using a stimulus set that dissociates taxonomy from face/body selectivity and from animacy as a binary distinction. Our findings reveal a very similar temporal profile for taxonomy and face/body selectivity with a peak around 150 ms. The binary animacy distinction has a more continuous and delayed temporal profile. These findings strengthen the conclusion that effects of animal taxonomy are in large part due to face/body selectivity, whereas selectivity for animate versus inanimate objects is delayed when it is dissociated from these other dimensions.

## Introduction

Vision is one of the senses that humans strongly rely on to obtain information about the world surrounding them. To create meaningful interactions with their environment, people subconsciously identify and categorize objects during everyday experience. Not only does the identification of objects occur without a second thought, it also happens in a remarkably fast fashion. Participants can decide very quickly whether a previously unseen natural image contains an animal, and object category representations in the brain emerge as early as 100 ms after stimulus onset (Carlson, Tovar, Alink, & Kriegeskorte, 2013; Thorpe, Fize, & Marlot, 1996). The superordinate categorization between animals and non-animals seems particularly important for human object vision and memory. Historically, neuropsychological studies in patients had already shown double dissociations between the knowledge about animals versus other objects (McCarthy & Warrington, 1994). Using modern brain imaging methods, object representations in occipitotemporal cortex (OTC) also show a tendency to cluster along an animacy distinction that separates animate from inanimate objects (Bao, She, McGill, & Tsao, 2020; Grill-Spector & Weiner, 2014; Kriegeskorte et al., 2008). When delving deeper into this animacy organization, researchers have suggested that it could reflect an animacy continuum or hierarchy more so than a binary animate-inanimate distinction (Sha et al., 2015; Thorat, Proklova, & Peelen, 2019). Object representations would then align themselves according to their level of animacy. Therefore studies investigating this functional OTC organization have built stimulus sets based on an intuitive animal taxonomy where different groups of animals can be ranked according to their level of animacy. For example, the mammal group would rank higher than the bird group, and even lower would be other groups like insects. This hierarchy is intuitive at the psychological level and very human-centered but not strictly biological. Research has shown that representational space in OTC can be understood in terms of this graded reflection of animacy, and is not based on a clear dichotomous distinction (Sha et al., 2015; Thorat et al., 2019). One way to interpret such findings would be that OTC represents semantic and conceptual properties of animals at a higher level of abstraction than visual features.

Object representations in the human brain are exceptionally rich (Bracci & Op de Beeck, 2023). On top of the animate-inanimate distinction and taxonomy, many other behaviorally relevant object properties are represented in OTC (Contier, Baker, & Hebart, 2024; Ritchie, Wardle, Vaziri-Pashkam, Kravitz, & Baker, 2025). Most prominent in the literature is the notion of category selectivity. Neuroimaging research on object processing has demonstrated robust selectivity for object category in OTC where specific areas respond to distinct categories, such as faces (Downing, Chan, Peelen, Dodds, & Kanwisher, 2006; Kanwisher, McDermott, & Chun, 1997; Puce, Allison, Asgari, Gore, & McCarthy, 1996), bodies (Downing, Jiang, Shuman, & Kanwisher, 2001; Downing et al., 2006), places (Downing et al., 2006; Epstein & Kanwisher, 1998), tools (Chao, Haxby, & Martin, 1999) and letters (Puce et al., 1996).

When investigating these multidimensional representations, we are confronted with the need to dissociate the dimensions from each other. In the current study we address this issue for the two highlighted dimensions: animal taxonomy and category selectivity, particularly face and body selectivity. The relationship between these two dimensions has not been given much attention. Indeed, studies investigating a possible taxonomic organization of OTC included stimulus sets that were not able to dissociate between both factors and compare the selectivity that each dimension could explain.

Theoretically, there are several possible relationships between the categorical face-body division at one hand, and the two variants of animacy representations: a binary animacy distinction and the more fine grained animal taxonomy. By definition, only animals have faces and bodies, providing an obvious relationship between faces and bodies on the one hand and the binary animal distinction on the other hand. Yet, within animals, the face-body distinction and the taxonomy factor could relate in very different ways, ranging from being two fully independent factors up to possibly being one and the same—where taxonomic effects would be caused by selectivity for faces and for bodies. To investigate how the face-body division and taxonomy relate to each other and contribute to the animacy organization of OTC, Ritchie et al. (2021) built a stimulus set that consists of different animals at different levels of the intuitive taxonomic hierarchy; and for every animal, a face and body picture. This way, neural patterns encoding these two factors could be disentangled from each other. The functional magnetic resonance imaging (fMRI) findings suggested that the taxonomic hierarchy found in OTC is a side effect of perceptual similarity to human faces and bodies. Most of the variance in multivariate similarity patterns was explained by this perceptual similarity to human faces and bodies, and typically no unique variance was additionally explained by taxonomic hierarchy. Faces and bodies of animals ranking higher in taxonomy have similar visual features to humans, which we know face and body regions are preferentially selective for. Thus the taxonomic organization at the neural level could be explained by the relative perceptual similarity of the stimuli to human faces and bodies.

However, the study of Ritchie et al. (2021), using fMRI did not consider the possibility that selectivity for these different factors might differ in the timing at which they emerge. Hemodynamic response patterns captured with fMRI provide useful information on the spatial distribution of neural activation patterns, but miss information about the temporal dimension. Yet, when object recognition takes place, information rapidly runs from low-level visual regions to higher-order areas where more integrated object representations emerge. To understand OTC organization and object processing, representational dynamics over time have to be taken into account (Cichy, Pantazis, & Oliva, 2014). By combining electroencephalography (EEG) with multivariate analyses and representational similarity analyses (Grootswagers, Wardle, & Carlson, 2017), we can investigate whether neural selectivity that appears to be linked spatially in fMRI emerges at similar time points or, alternatively, would be dissociated temporally.

In the present study, we investigated the temporal dynamics of the three aforementioned distinctions—the face-body division, animacy, and taxonomy—to understand how the representations emerge and differ between conditions that vary in their level of abstraction. The stimulus set of Ritchie et al. (2021) provided an opportunity to do so, and we performed a decoding analysis on data from high-density EEG to capture the temporal dynamics of all three factors of interest. Additionally, with the fMRI dataset from Ritchie et al. (2021) we had the possibility to perform fMRI-EEG fusion where similarity of neural representations between both imaging modalities is compared to reveal the common spatiotemporal dynamics.

## Material and methods

### Participants

Twenty-five healthy volunteers (18 females, mean age 23 years) were recruited through the recruitment system (SONA) of the Faculty of Psychology & Educational Sciences at KU Leuven, and participated for monetary compensation. The experiment was approved by the Social and Societal Ethics Committee (SMEC) at KU Leuven (G-2019 12 1888). Volunteers provided their written informed consent before participating and were fully informed about the experimental task and the EEG procedure before starting.

### Stimuli

**Figure 1.**
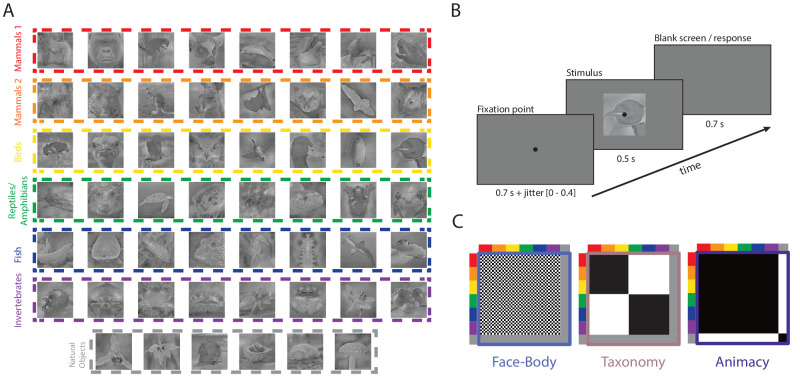
Experimental design. (**A**) The 54 stimulus conditions, with 24 animals represented each by a body and a face image, supplemented with six object images. Images can be viewed individually at higher resolution and downloaded from https://osf.io/xcpw6/files/osfstorage. (**B**) The structure and timing of a trial in the EEG experiment. (**C**) Visual display of the three binary divisions of the stimulus set: Face/body (the 24 face images vs. the 24 body images), Taxonomy (the 24 animals at the highest taxonomic levels vs. the other 24 animals), and Animacy (the 48 animals vs. the six natural objects).

The stimulus set used for this experiment was previously constructed by Ritchie et al. (2021) and consisted of 54 images (Figure 1A). 24 animals were depicted, each with a face and body picture. The stimulus set was built in such a way as to represent an intuitive taxonomic hierarchy where the animal images cluster into six levels of taxonomy: mammal cluster 1, mammal cluster 2, birds, reptiles/amphibians, fish, and exoskeletal invertebrates. This choice of taxonomic levels is based on the earlier literature finding taxonomy effects (e.g., Connolly et al., 2012; Sha et al., 2015) and is further motivated in Ritchie et al. (2021). In addition, there were six natural object images, from the categories of orchids, fruit/vegetables, and mushrooms. Further processing, also taken from Ritchie et al. (2021), included cropping all images to 700 × 700 pixels, blurring the background region of each image, converting the images to grayscale and equalizing the luminance histogram with the average energy of each spatial frequency with the SHINE toolbox (Willenbockel et al., 2010).

### Naming task before EEG experiment

Following the design of Ritchie et al. (2021), participants were asked to perform a naming task prior to the EEG experiment to ensure their ability to recognize all stimuli during the upcoming EEG session (Figure 1A). A fixation cross was presented for 500 ms, after which a randomly selected stimulus appeared for 1000 ms. Participants were then asked to type the correct name of the animal/natural object either in Dutch or English and press “enter,” after which the correct name appeared in both languages for 3000 ms. If the stimulus was recognized but the participants could not provide the correct name, they were instructed to press “y.” If the stimulus was unknown to them, they were instructed to press “n.” The answers were then compared to predetermined labels to calculate correct responses. Stimulus presentation and response collection were executed using PsychoPy2 (Peirce, 2007) on a Windows laptop.

### Experimental task during EEG experiment

Participants were instructed to focus on the fixation point that was presented before stimulus onset (Figure 1B). The fixation point had a presentation time between 700 and 1100 ms to reduce any expectation effects. Afterwards a randomly chosen stimulus would be presented for 500 ms, followed by a 700 ms blank screen. As with the study of Ritchie et al. (2021), during the blank screen (and not earlier nor later), participants had to answer whether they liked looking at the image or not by pressing either the left or the right arrow. After 700 ms, the next trial started, and the fixation point appeared again. Yes and no buttons were counterbalanced in each run, and always clearly stated at the beginning of the next run. Each participant completed 10 runs that in total lasted approximately 40 minutes. For two participants, one of the runs was not successful, so the total amount of runs for these volunteers was 9. Stimuli were presented in random order during a run, and each stimulus was presented twice per run, resulting in 108 trials per run. Stimulus presentations and response collection were executed using the Psychophysics Toolbox package (Brainard, 1997) in Matlab (The MathWorks Inc, 2022) in combination with custom code. For the experimental task the participants were seated in a dark testing cubicle, approximately 50 cm away from the computer screen. The researchers were seated in an adjacent room, with a view of the testing cubicle and communication possible through the intercom.

### EEG procedure

#### EEG acquisition

EEG signals were recorded with a high-density 128-channel system (Philips Electrical Geodesics Inc., Eugene, OR, USA) using a sampling rate of 1000 Hz. The exact stimulus onset was tracked with a photocell sensitive to contrast changes. The photosensor was placed at the bottom of the screen where a small square appeared together with the stimulus and recorded the exact timing of stimulus onset.

#### EEG preprocessing

Offline preprocessing of the EEG data was done with the Fieldtrip toolbox (Oostenveld, Fries, Maris, & Schoffelen, 2011) in Matlab (The MathWorks Inc, 2022). For every participant, bad channels were identified by visual inspection and impedances exceeding 1000 Ohm. These channels were later excluded from analysis. The preprocessing pipeline included a high pass filter of 2 Hz to remove slow drifts, and a bandstop filter of 50 Hz to remove powerline noise and its harmonics. Afterwards, data was re-referenced to the average, baseline corrected, and resampled to 250 Hz.

To remove noise components, we performed an independent component analysis (ICA) in EEGLAB (Delorme & Makeig, 2004) with the ICLABEL plugin (Pion-Tonachini, Kreutz-Delgado, & Makeig, 2019). This plugin runs an algorithm that labels independent components with one of seven tags: brain, heart, muscle, eye, line noise, channel noise, other. Every labeled component can be further investigated by opening its specific interface that shows the topography of the component, the time course, power spectrum and event-related potentials (ERP) image. Based on the labels and a visual inspection of its details, we excluded eye components (including eyeblinks and eye movements), muscle components, line noise and heart noise (if present). After exclusion of the noise components, the EEG signal is reconstructed based on the remaining components and put back into a fieldtrip structure for further processing. In some isolated cases, ICA did not produce a meaningful decomposition and every component was labeled as a noise component. For these runs, we refrained from ICA and kept the original signal. After ICA, the continuous signal was segmented into corresponding trials, ranging from −400 ms to 500 ms relative to stimulus onset, resulting in 108 trials of 900 ms per run per participant.

#### Categorical decoding analysis

Multivariate pattern analysis (MVPA) was implemented to detect differential neural activity related to binary categorical distinctions. Three distinctions were considered: Face/body, Taxonomy, and Animacy (Figure 1C). For the face/body distinction, only the face and body images of all animals were used, with 24 images in each category. The taxonomy distinction was defined by splitting up the animal group in such a way as to contrast high versus low taxonomy with an equal number of 24 animals per level (mammals 1, mammals 2 and birds vs. reptiles/amphibians, fish, and invertebrates). This approach also results in 24 images in each category. For the animacy distinction, we separated the 48 animal from the six natural object stimuli. This resulted in an uneven sample size for both groups. To eliminate any potential biases in the decoding of the animacy distinction, the partitions for training and testing were balanced so that every condition label occurs equally often in both.

For the decoding analysis, code was written in Matlab (The MathWorks Inc, 2022) using the CosMoMVPA toolbox (Oosterhof, Connolly, & Haxby, 2016), and adapted from the code of Chen, Leys, Bracci, and Op de Beeck (2023). We implemented a temporal searchlight analysis. A linear discriminant analysis classifier was trained and tested using leave-one-run-out cross-validation. The classifier was trained to distinguish between both options within every condition, including all trials from all runs but one. Testing of the classifier was done on the individual trials of the left-out run. This procedure was repeated until every run was left out once. All 128 channels were included for the searchlight analysis. The temporal neighborhood was set up with a radius of 2, resulting in a window of 5 time bins, one center time point and four neighboring time points. A searchlight analysis was performed for every condition and participant separately, giving us decoding accuracies over the full trial period.

#### Scalp topography

A searchlight analysis in space and time was performed in CosMoMVPA for each condition and participant to localize object category related information in the EEG signal. For every sensor, a different spatial neighborhood was constructed that included the corresponding sensor and its nine nearest neighbors, based on the spatial template of the GSN Hydrocel 128 channel net. After running the searchlight analysis over all 128 channels and all time points, we obtained a topography of decoding accuracies over time, which was averaged across participants.

#### Representational similarity analysis (RSA)

Decoding analysis was also implemented to investigate the neural similarity of individual stimulus pairs over time. We ran a searchlight analysis, with the same settings as the categorical decoding, but now for each stimulus pair separately. The result was a 54 × 54 representational dissimilarity matrix (RDM) for every time point where each pairwise decoding accuracy represents a measure of neural dissimilarity between stimuli. For comparison between the model and EEG RDMs, only the top half of the matrices was used. Individual EEG matrices were correlated with the model matrices for each timepoint and correlational values were then averaged across participants to reflect the similarity between the neural representational dynamics and model RDMs.

First, we compared the EEG matrices with three theoretical model RDMs derived from the three aforementioned categorical distinctions of interest: 0 versus 1 for face versus body; 0 versus 1 for the low versus high taxonomic levels (high = the 24 mammal and bird stimuli), 0 versus 1 for animate versus inanimate. The final model RDMs contain the absolute difference in the pairwise values of the corresponding stimuli (Figure 1C). The face/body and the taxonomic level RDM were only defined and applied for the 48 animal stimuli, thus all RSA analyses for these models are based on these 48 stimuli. An additional RDM was modeled based on global image structure tensor (GIST) descriptor. Every image was partitioned into a 4 × 4 grid, and for each block in the grid, Gabor filters (eight orientations and four spatial frequencies) were applied. Next, the values of each filter were vectorized and the final RDM contained the pairwise 1 − *r* Pearson's correlations between the vectors.

Next, we compared the EEG matrices per timepoint with RDMs from the fMRI experiment of Ritchie et al. (2021) in several regions of interest (fMRI-EEG fusion). Note that the fMRI and EEG experiments included different participants. The fMRI RDMs were built using the (non-cross-validated) Mahalanobis as a metric for dissimilarity reflected by the pairwise distance along the discriminant between conditions for the beta weight patterns in an region of interest (ROI) (Ritchie & Op de Beeck, 2019; Walther et al., 2016). Seven ROIs were decided upon by Ritchie et al. (2021): lateral occipitotemporal cortex (LOTC)-body, LOTC-face, LOTC-object, ventral occipitotemporal cortex (VOTC)-BODY, VOTC-face, VOTC-objects and early visual cortex EVC. Detailed information about the ROI definition, can be found in Ritchie et al. (2021). Briefly, the LOTC and VOTC subregions were defined using an independent localizer. These ROIs contain all voxels with significant activation in the contrast of bodies minus (faces + objects) for body regions and faces minus (bodies + objects) for face regions. EVC was defined anatomically as the posterior (most foveal) part of the V1 mask as defined in the Anatomy Toolbox in SPM. Once again, only the top half of the matrices were used for RSA. Individual EEG RDMs were correlated with averaged fMRI matrices for every timepoint and for every ROI and afterwards averaged across participants, providing a comparison between neural activity of different functional regions in the brain and temporal dynamics of the same stimulus set.

#### Statistical inference

To check for statistical significance of our decoding analysis, we implemented a threshold-free cluster enhancement procedure (Smith & Nichols, 2009) as provided in CosMoMVPA. Adjustments for multiple comparisons were carried out by constructing null distributions filled with the results of 1000 bootstrapping iterations. Significance of decoding accuracies was assessed against chance level of decoding (50%) with permutation tests where category labels were randomly assigned 100 times for every participant. Significance threshold was set at *p* < 0.05 with a two-tailed test (*z* > 1.96 and *z* < −1.96).

## Results

### Decoding analysis

**Figure 2.**
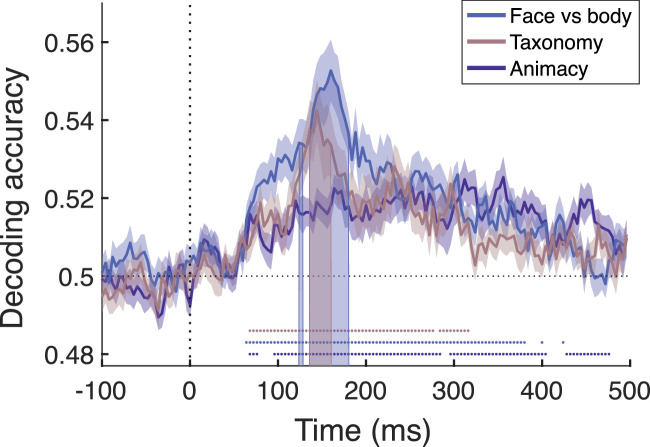
Decoding accuracy in the temporal searchlight analysis for the three distinctions of interest: Face/body, Taxonomy, and Animacy. Accuracy was averaged across participants, and the shaded area around the data lines represent the standard error of the mean across participants. The vertical dotted line represents stimulus onset, whereas the horizontal dotted line is placed on 0.5 or chance-level of decoding accuracies. The dots underneath the baseline represent significant timepoints for each corresponding distinction. Shaded bars represent significant differences between Animacy and the other two distinctions. All statistics are corrected for multiple comparisons across time points.

We used a stimulus set that allowed us to separately investigate the representational dynamics of different distinctions, including the binary distinctions of faces versus bodies, animate versus inanimate (Animacy), and animals that are high versus low on taxonomy. This provides three categorical distinctions, Face/body, Animacy, and Taxonomy. We performed a decoding analysis on multi-sensor response patterns for each distinction. We used a temporal searchlight method in which a decoder is trained and tested using response patterns in temporal neighborhoods around specific time points and the time window moves from before stimulus onset up to stimulus offset. Figure 2 shows the decoding accuracy over time for every distinction, averaged over all participants.

Decoding accuracy of all three distinctions simultaneously rises from around 60 ms after stimulus onset. For both Face/body and Taxonomy peak decoding accuracy was achieved around 150 ms, after which decoding gradually declines. Highest decoding accuracy can be found for Face/body, which indicates that the neural signals involved in face versus body processing are easiest for the classifier to pick up on. For the Face/body distinction, decoding remains significant until about 400 ms, after which it returns to chance level. Taxonomy is associated with a similar temporal profile, but because of its lower overall decoding performance, it shows a faster decay of decoding with its significance disappearing around 320 ms. The Animacy distinction in general has a more constant decoding time course, without a clear peak, but remains significant and rather consistent for almost the whole trial. This absence of a peak accuracy for Animacy is also reflected in significant differences between Animacy and the other two categories during the peak accuracy period (shaded area in Figure 2). When comparing the decoding time courses, the decoding for Animacy is significantly lower than for both Face/body and Taxonomy in the time interval of 124–180 ms. This finding contrasts with the situation later in the response, after 400 ms, where Animacy is the only distinction that is still showing significant decoding.

**Figure 3.**
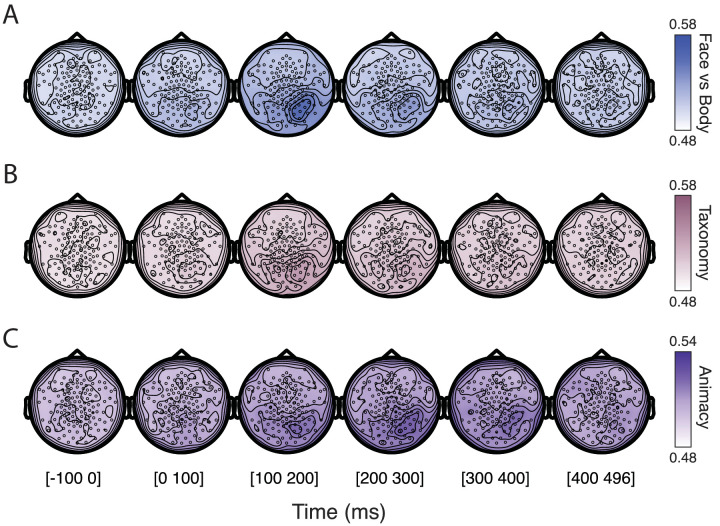
Spatiotemporal searchlight analysis results for all three conditions of interest: (**A**) Face/body, (**B**) Taxonomy, and (**C**) Animacy. The figure shows broadly the spatial configuration of the source activity for decoding, separated in six segments of 100 ms representing the evolution of decoding activity over space and time. Note that to enhance visibility of spatial topography, we rescaled the decoding activity for Animacy to 0.48–0.54, which is different from the range of Face/body and Taxonomy, that both span 0.48–0.58.

A spatiotemporal decoding analysis was also performed to identify the source of decoding activity in sensor space. This searchlight iterates over different spatial neighborhoods together with the previously specified temporal neighborhoods. The outcome provides a rough localization of the spatial topography that underlies the decoding in different time intervals. Based on previous fMRI research, we expected the object-processing regions to represent all the different distinctions in our stimulus set, therefore the sensors covering OTC are expected to be involved. Figure 3 shows the spatial maps of neural activity for every distinction separately. For all three distinctions we see that the highest decoding involves the right posterior electrodes, which broadly correspond to OTC. The strongest activity can be seen for Face/body between 100 and 200 ms which is the period of peak decoding accuracy, and seems to be restricted to the neighborhoods of a few sensors. In later time points, the decoding activity gradually declines. Temporal dynamics of Taxonomy appear to have a similar topography and timing as Face/body, with the 100-200 ms period indicating strongest decoding that disappears over time. Animacy decoding again shows a similar spatial topography as the other two conditions, with sensors covering (right) OTC contributing to the higher-than-chance-level decoding. However, this activity is more spread out in time, only going down to chance level at the end of the trial, and maybe more spread out over the posterior electrodes.

Overall, the decoding analyses support the conclusion that all three distinctions are based on signals with a similar spatial topography, as far as such conclusions can be reached with EEG data. In terms of temporal dynamics, the Animacy distinction stands apart from Face/body and Taxonomy because it does not show a strong peak in decoding relatively early on and has a steadier level of decoding up to later time points.

### Representational similarity analysis for pairwise stimulus differences

**Figure 4.**
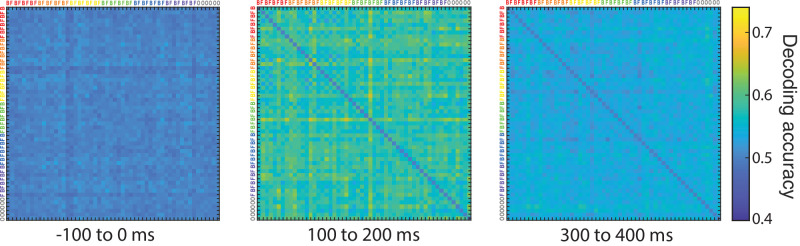
RDM matrices for all individual stimuli, pictured for three time segments with decoding activity averaged per segment; pre-stimulus onset, 100–200 ms after stimulus onset and 300–400 ms after stimulus onset. On both axes we can see annotations for all images; colors indicate different levels of taxonomy (mammal cluster 1 in red, mammal cluster 2 in orange, birds in yellow, reptiles/amphibians in green, fish in blue, exoskeletal invertebrates in purple, and inanimate objects in gray), face and body images of the animate objects are annotated with, respectively, an F or a B, inanimate objects are referred to by an O. The diagonal is set to 0.5 for all stimuli.

The aforementioned searchlight analysis can also be performed on individual images to capture pairwise differences between stimuli for every timepoint. Every stimulus is decoded against every other stimulus giving us an indication of the neural similarity between stimuli and how this evolves over time. The resulting decoding accuracies can be placed into a representational dissimilarity matrix (RDM) with the stimuli placed along both axes so that the matrix is mirrored along the diagonal, offering a visualization of the dissimilarity between individual stimuli. Figure 4 shows RDMs containing pairwise representational differences for three different time intervals providing a rough picture of how these representations emerge and change over time. The time interval before stimulus onset (−100 to 0 ms) shows decoding accuracies around chance level because there is not yet decodable information in the data. In later time points we see that decoding accuracies rise above chance level, such that 100 to 200 ms after stimulus onset neural patterns for individual stimuli are different enough from each other for reliable decoding to occur. The last panel in Figure 4 shows a degradation of the decoding, with accuracies going back down.

**Figure 5.**
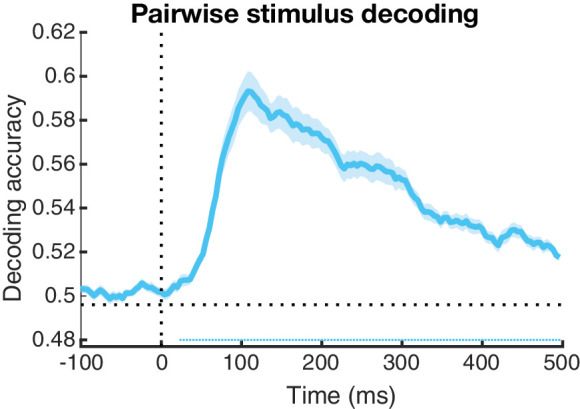
Time course of decoding activity when comparing individual stimuli. Accuracy was averaged across stimulus pairs and across participants. The shaded area around the data line represent the standard error of the mean across participants. The horizontal dotted line is set on 0.5 and represents chance level, the vertical dotted line represents stimulus onset at 0 ms. Decoding accuracies significantly higher than chance level, after correction for multiple comparisons, are depicted by the dotted line below chance level.

To assess the temporal profile of overall pairwise decoding in more detail, we averaged the aforementioned RDMs across all stimuli and all participants for every timepoint. As seen in Figure 5, decoding accuracy is higher (peak close to 60%) on an individual stimulus level and peaking earlier (around 100 ms) than when decoding was done for broader distinctions (as was shown in Figure 2). Even though decoding on an individual image level has less training data to work with compared to condition-based decoding, the classifier can work with features specific to the individual images, simplifying the classification process. When grouping stimuli according to a categorical distinction, individual differences between images can no longer be used as a classification help, but instead add additional noise for the classifier that is now forced to find the signals that are common among stimuli. Therefore a higher decoding accuracy for pairwise differences is to be expected. This could also explain why the peak of decoding is earlier than in the previous analyses.

### Representational similarity to theoretical models

**Figure 6.**
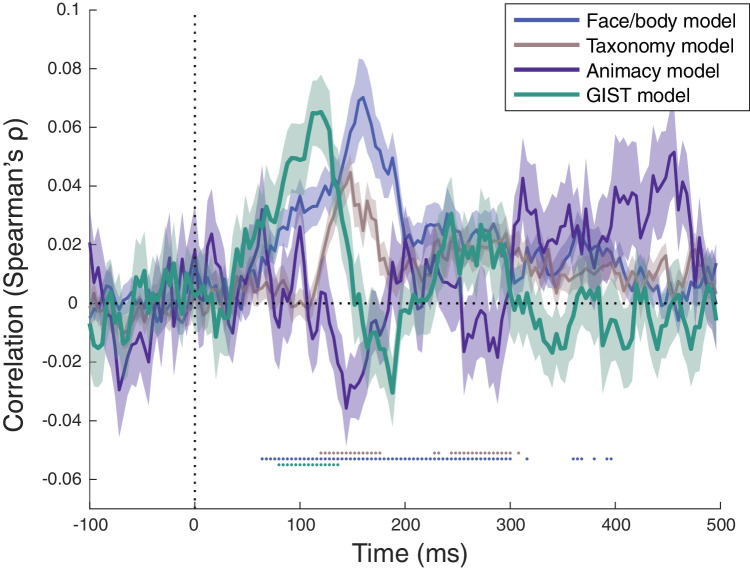
Time course of the correlations of the EEG data with the theoretical models. Accuracy was averaged across participants, and the shaded area around the data lines represent the standard error of the mean across participants. The horizontal gray dotted line represents correlation of zero, the vertical gray dotted line represents stimulus onset at 0 ms. Significant time points, after correction for multiple comparisons, are depicted by dotted horizontal lines below the zero-correlation line with the colors corresponding to the different models.

Theoretical model RDMs were constructed in line with how we divided the stimulus set in the decoding analysis, resulting in a binary model RDM for Animacy, Face/body and Taxonomy. An additional GIST model RDM was constructed, based on low-level features of the images. These model RDMs were next correlated with the EEG matrices of every participant individually for every timepoint, and then averaged across all participants (Figure 6). Given that Face/body and Taxonomy are not defined for the six inanimate stimulus conditions (which were also not included in the decoding of these distinctions), these models and the computations on the EEG matrices included only the 48 animate stimulus conditions. This approach also avoids the small confounding between these two model matrices and the Animacy model that would otherwise have existed.

Early timepoints, with a peak around 100 ms, show a significant correlation with the GIST model. This is expected given that the GIST model is thought to capture the processing of simple image features. At later time points, we find strong correlations with the Face/body model. These correlations are significant from very early already, show a peak around 150 ms, and stay up until at least 300 ms after stimulus onset. This temporal profile seems consistent in general with what we observed in the decoding analysis. The same goes for correlations with the Taxonomy model. Its temporal profile is similar to the profile of the Face/body model, although clearly weaker than the correlations for Face/body.

The Animacy model reveals a very different profile, with higher values only in the last 200 ms of stimulus presentation. Although this temporal profile of correlations with the Animacy models shows extreme values in the same range as for the Taxonomy model, none of these individual time points reaches significance after the threshold-free cluster enhancement correction for multiple comparisons. The estimates for the Animacy model might be less reliable because we have far less data for the inanimate condition, with only six stimuli. Our experimental design puts this distinction at a disadvantage compared to the other models. In addition, decoding is worse in general at later time points (see Figure 5), which is also detrimental for a model whose correlations are emerging later. Still, the difference in the temporal profile relative to the other conditions of interest is striking and extended over time.

For this reason, we explicitly tested whether the Taxonomy model and the Animacy model significantly differ in their time course. We averaged the RDMs in the interval 100–300 ms and the interval 300–500 ms, where 300 ms marks the time where the correlations with the non-involved model (Face/body model) become nonsignificant. Testing this interaction pattern in full, the time interval (early vs. late) interacted significantly with model (Animacy vs. Taxonomy), (*F*(1,24) = 52.1462, *p* < 0.0001. Further explorative *t*-tests show that for the Animacy model there is a consistently larger correlation in the later time interval compared to the earlier interval (*t*(24) = −4.4060, *p* = 0.0002), whereas the opposite is observed for the Taxonomy model (*t*(24) = 2.58, *p* = 0.0163).

### Representational similarity to fMRI data

Next, we performed fMRI-EEG fusion to compare representations between both imaging modalities. Previous work by Ritchie et al. (2021) compared activity between different visual brain regions, resulting in RDMs for seven ROIs: bilateral LOTC-body, LOTC-face, LOTC-object, VOTC-BODY, VOTC-face, VOTC-objects and V1. Here, we correlate the off-diagonal values in the RDMs of these ROIs as obtained with fMRI with the values of the RDM of the EEG at each time point. This EEG RDM contains the pairwise decoding differences using all EEG sensors, same as in the analyses reported in the previous sections.

**Figure 7.**
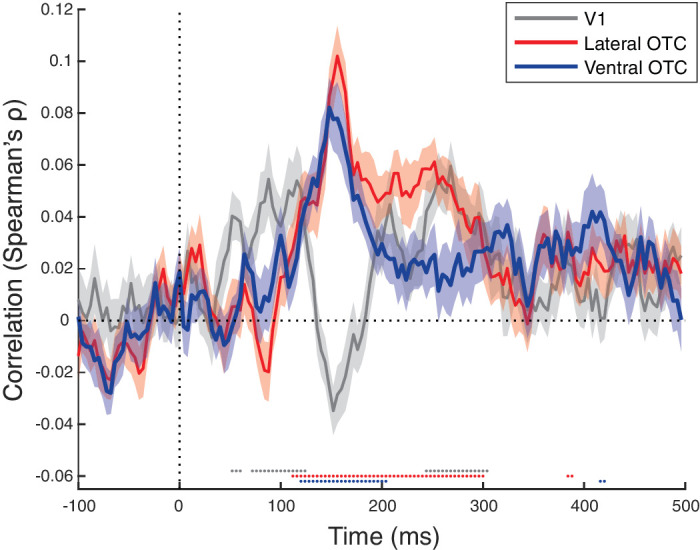
Time course of the correlations of representational dissimilarity (pairwise decoding matrix) in the EEG data with the representational dissimilarity in fMRI data. Accuracy was averaged across participants, and the shaded area around the data lines represent the standard error of the mean across participants. Horizontal gray dotted line represents correlation of zero, the vertical gray dotted line represents stimulus onset at 0 ms. Significant time points, after correction for multiple comparisons, are depicted by dotted lines below the zero-correlation line with the colors corresponding to the different brain regions.

First, we combined the subregions of OTC to correlate with our EEG data; lateral OTC included all the lateral subregions, ventral OTC all the ventral ones. Figure 7 shows the correlation time course of V1, lateral OTC and ventral OTC with our EEG data. We see an early emergence of correlations with V1, about 50 ms after stimulus onset, which steadily increases and reaches a peak around 100 ms. Next, we see a very sudden drop of the V1 correlation while at the same time both lateral and ventral OTC show a strong rise followed by a peak around 150 ms. Ventral OTC drops down to nonsignificance quite soon after its peak, whereas lateral OTC remains more steady until 300 ms where it further declines to nonsignificance. Interestingly, we see a re-emergence of V1 correlations, between 250 and 300 ms, to a similar level as lOTC. Such rebound effects have previously been taken as a reflection of recurrent processing (Goddard, Carlson, Dermody, & Woolgar, 2016; Kietzmann et al., 2019).

**Figure 8.**
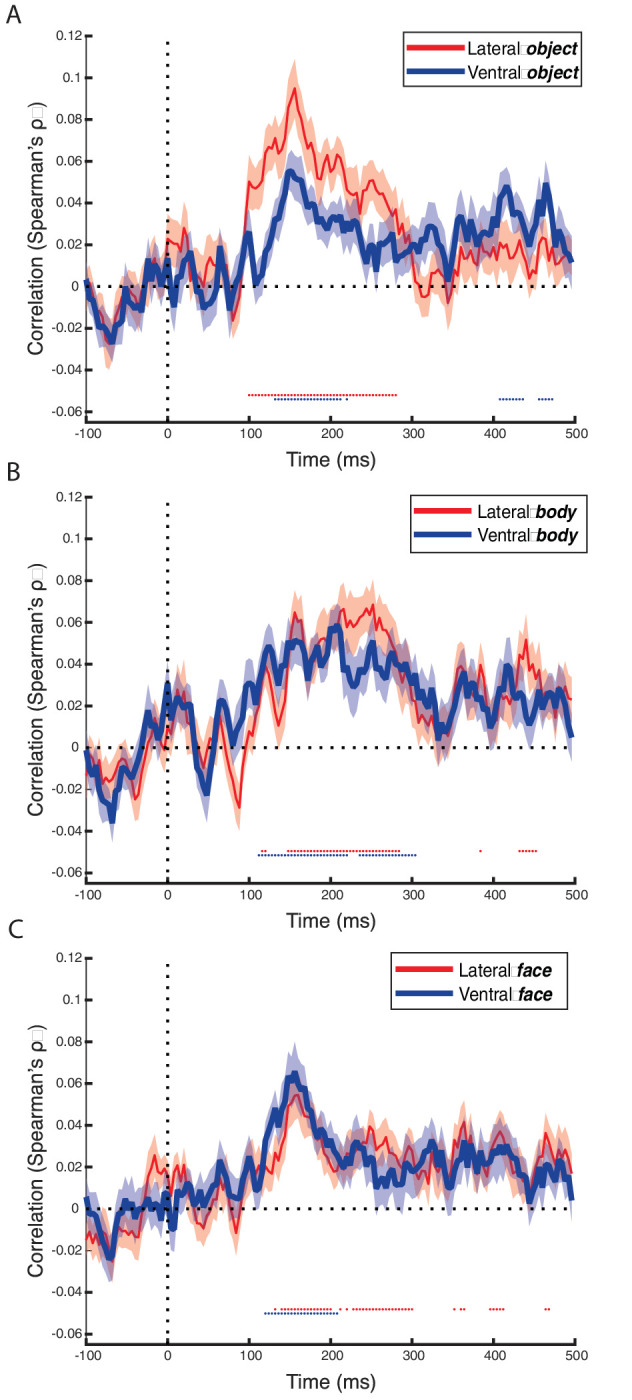
Time course of the correlations of the representational dissimilarity (pairwise decoding matrix) in EEG data with the representational dissimilarity in fMRI data from different subregions: (**A**) object-selective regions, (**B**) body-selective regions, and (**C**) face-selective regions. Lateral regions are depicted in red, ventral regions in blue. Accuracy was averaged across participants, and the shaded area around the data lines represent the standard error of the mean across participants. Horizontal gray dotted line represents correlation of zero, the vertical gray dotted line represents stimulus onset at 0 ms. Significant time points, after correction for multiple comparisons, are depicted by dotted lines below the zero-correlation line with the colors corresponding to the different brain regions.

Additionally, we correlated our EEG data with all OTC subregions separately (Figure 8). Overall, the correlations copy the pattern of all of lateral and ventral OTC, with no large differences between the nearby subregions. Strongest correlations can be found between 100 and 200 ms after stimulus onset, with most of the peaks around 150 ms. All subregions appear to be equally involved in representational dynamics of our stimuli. This is consistent with the findings from Ritchie et al. (2021) that a similar coding scheme is present in all these regions.

## Discussion

We investigated the timing at which three different object distinctions are activated: faces versus bodies, taxonomic level of the depicted animals, and animacy (animate or not). We found a striking similarity between the temporal profile of the face/body distinction and of the taxonomic level, peaking around 150 ms and falling down afterwards. In contrast, animacy representations showed a different temporal profile with stronger representations after 300 ms. These representations were broadly localized to right OTC regions in the EEG data. The fMRI-EEG fusion analysis showed that correlations with representations in lateral and ventral occipitotemporal regions show a similar temporal profile to the face/body selectivity and taxonomic selectivity.

A previous fMRI study with the same stimulus set concluded that the effects of taxonomy in fMRI data can be traced back to how faces and bodies are represented (Ritchie et al., 2021). Faces and bodies of animals are represented in terms of how similar they are to human faces and animals (see also Contini, Goddard, Grootswagers, Williams, & Carlson, 2020). This explains apparent taxonomy effects because faces and bodies of animals that are considered more animate (e.g., mammals) are perceptually more similar to human faces and bodies than faces and bodies of less-animate animals (e.g., insects). From this hypothesis we would also expect that the face/body distinction and taxonomy effects should have similar temporal profiles. This was confirmed by the present study in the decoding analysis as well as in a representational similarity analysis. Overall, the present findings strengthen the evidence that taxonomic effects related to “degree of animacy” are due to how faces and bodies are represented.

In contrast, the temporal profile was different for the binary animacy distinction. The representational similarity analysis shows the strongest dissociation between these temporal profiles, with a very late rise in selectivity for animacy. In the decoding analysis the selectivity for animacy emerges earlier and remains more or less constant towards later time points. In previous studies, the representational dynamics related to animacy were also earlier (Carlson et al., 2013; Cichy et al., 2014; Kietzmann et al., 2019). Not as early and with as prominent a peak around 150-200 ms as what we find for face/body and taxonomy, but more so than what we find with the current paradigm. This might be related to the characteristics of our stimulus set, and in particular that it dissociates taxonomy from face/body and from animacy. As a side effect, our stimulus set contains a different distribution of stimuli across taxonomic levels. The most common stimulus set, first used by Kriegeskorte et al. (2008) and adopted by Cichy et al. (2014) and Kietzmann et al. (2019), contains a lot of mammals as animals, and as a consequence a lot of human-like bodies and faces. As a consequence, the human similarity effect that underlies face/body and taxonomy selectivity according to Ritchie et al. (2021) can strongly contribute to the animacy representations with the stimulus set of Kriegeskorte et al. (2008). From this perspective, the current study is a strong motivator for future studies to tease apart the multiple factors that might often correlate with animacy, as the failure to tease these dimensions apart can strongly impact their representational dynamics.

One previous study dissociated two other dimensions related to animacy, visual categorizability and agency, and showed that these two dimensions were associated with different regions in occipitotemporal cortex, with more posterior selectivity for categorizability (Thorat et al., 2019). With our current stimulus set, Ritchie et al. (2021) did not report a different topography for taxonomy and animacy, nor is there any indication for a difference in our current EEG study. With taxonomy and animacy, the dissociation is more obvious in time than in space, with much later representational dynamics for animacy compared to taxonomy.

Jozwik et al. (2022) reported the most comprehensive and recent investigation of the interplay between, and timing of, multiple high-level dimensions related to animacy, including dimensions like “being alive,” “looking like an animal,” “having agency,” “having mobility,” and “being unpredictable.” The strongest correlation with EEG patterns was observed for the dimension “looking like an animal.” This is the dimension that in their study most consistently contrasts all the images that contain faces and bodies from images that do not. This fits with our suggestion that a lot of animacy-related findings in the literature might be due to perceptual similarity to faces and bodies. Yet, the timing of this correlation in their study is different (later) from ours, nor did they differentiate between faces and bodies.

Returning to the topic of the representation of animacy that we observed at later time intervals, we can only speculate which processes underlie this representation and how it comes about. Our design is configured to tease it apart from taxonomy and face/body selectivity, but not to investigate the details of this animacy representation with high conceptual precision. An obvious problem is that our inanimate stimuli included only six exemplars. A comparison with Jozwik et al. (2022) is not very informative, given that none of their dimensions showed a temporal profile that is similar to what we found for animacy in Figure 6. Yet, they do not have any dimension that would really set apart all animals from all non-animals, which is how we defined animacy. Possibly, this binary semantic distinction, independently from its more perceptual and gradual correlates of “looking like an animal/body/face,” is driving the later responses in higher levels of the visual system. More work will be needed to connect all the dots and arrive at an integrated understanding of the relationship between perceptual and cognitive factors underlying animacy representations and how they relate across brain regions and across time.

Our findings from the fMRI-EEG fusion analysis are expected given what was found in the previous analyses (decoding and RSA). Ritchie et al. (2021) showed that all three models, Face/body, Taxonomy, and Animacy, explain part of the multi-voxel similarity patterns in ventral and lateral occipital cortex, with the strongest correlations for face/body. Ventral and lateral regions show very similar representations in fMRI, and here we show that they also converge in terms of the timing at which the representations in these two sets of regions correlate with the EEG. This correspondence between fMRI and EEG in the degree to which these different models explain the neural data suggests that these imaging modalities are not differentially biased in favor or against particular levels of representation. That is not a trivial finding, giving that in at least one previous comparison MEG was shown to be less sensitive to animacy representations compared to what would be expected from fMRI (Proklova, Kaiser, & Peelen, 2019).

The temporal profile of correlations between time-wise EEG representations and fMRI activity contributes to a consistent picture in our case. Given that both face/body and taxonomy have a peak in temporal decoding profile around 150 ms, we can expect that fMRI/EEG fusion would show, as it did, that the fMRI similarity patterns from these brain regions would explain the EEG data best around these timepoints. Primary visual cortex was the only region analyzed by Ritchie et al. (2021) that showed a different representational content, with a strong correlation with the GIST model but without any multivoxel similarity patterns related to face/body, taxonomy, or animacy. From these fMRI findings it was fully predictable that the fMRI/EEG correlation for V1 in Figure 7 would appear at similar timepoints as the significant correlations with the GIST model in the EEG RSA analysis shown in Figure 6.

Taken together, the current findings illustrate how we can understand the relationships between prominent dimensions of object representations by combining clever stimulus designs with both fMRI and EEG. More specifically, we provide evidence that part of the selectivity that is often associated with animacy, more specifically the effects of the taxonomic level, seems due to face/body selectivity of object representations, both in terms of multi-voxel selectivity patterns in fMRI (Ritchie et al., 2021) and in terms of temporal profiles (current findings). In the literature, selectivity for animal taxonomy and for faces versus bodies has been studied separately, and are often treated as separate phenomena. Previous findings related to the apparent animacy taxonomic continuum were also interpreted in different terms, with reference to a coding of semantic and conceptual properties in case of animal taxonomy and typically more perceptual interpretations in case of face/body selectivity (e.g., Jozwik et al., 2022; Thorat et al., 2019). Yet, based on our findings, we argue that the two dimensions might have a common origin, reflecting the perceptual similarity of stimuli to human faces and bodies. At the same time, other aspects of animacy, in the present case the binary distinction between animate and non-inanimate, are distinct in terms of temporal profile, despite obvious overlap in terms of where selectivity is found according to fMRI, and can show a very different temporal profile once they are properly dissociated from the other dimensions. The lower sensitivity of fMRI relative to EEG for this binary animacy distinction might explain why Sha et al. (2015) found in their fMRI study that there was no such dichotomous distinction. The present study, combined with the earlier experiment of Ritchie et al. (2021), highlights the relevance of testing experimental designs with both fMRI and EEG to obtain a complete spatiotemporal characterization of how representations unfold across brain regions and across time.
